# Global research trends on the relationship between gut microbiota and melanoma from 2014 to 2023: a bibliometric and visualization analysis

**DOI:** 10.3389/fmicb.2025.1524462

**Published:** 2025-08-13

**Authors:** Sai Liu, Kewang Hu, Mingli Zhu

**Affiliations:** Hangzhou Xixi Hospital, Hangzhou Sixth People's Hospital, Hangzhou Xixi Hospital Affiliated to Zhejiang Chinese Medical University, Hangzhou, Zhejiang, China

**Keywords:** bibliometrics, melanoma, gut microbiota, immunotherapy, VOSviewers, citespace

## Abstract

Melanoma, a type of malignant tumor, originates from skin cells known as melanocytes. The gut microbiota is closely linked to melanoma, and this bibliometric analysis aims to guide future studies and advance research into the role of the gut microbiome in melanoma. We used the Web of Science Core Collection database to identify relevant literature on the function of intestinal flora in melanoma from 2014–2023, and bibliometric analysis and network visualization were performed with VOSviewer, CiteSpace. A total of 886 articles were identified, with significant contributions from the United States, France, and China. The role of gut microbiota in the onset, progression, and treatment of melanoma is becoming increasingly clear, especially in its potential to improve immunotherapy outcomes. In conclusion, this research used bibliometric analysis to perform a thorough quantitative and qualitative evaluation of the literature concerning the role of gut microbiota in melanoma. Advancing research in this area offers hope for enhancing melanoma treatments by regulating the gut microbiome, which could lead to improved patient outcomes in the future.

## Introduction

Microorganisms in the gut include bacteria, fungi, viruses, and various other microbes (Kuziel and Rakoff-Nahoum, 2022). It is estimated that the human body contains approximately 4 × 10^13^ microbial cells, the majority being bacteria, with over 95% residing in the gut (Sender et al., 2016). The gut microbiota plays a crucial role in regulating human metabolism, development, and the immune system (Knights et al., 2013). Moreover, the microbiome has garnered significant attention for its role in various human diseases, including cancer (Heintz-Buschart and Wilmes, 2018).

Melanoma is a malignant tumor that originates from the melanocytes of the skin (Long et al., 2023), Melanoma of the skin causes 55,000 deaths a year and can quickly become life-threatening once the disease has spread (Schadendorf et al., 2018). The occurrence and death rate of melanoma vary significantly across different regions of the world. In Europe , the occurrence of melanoma is about 25 new cases per 100,000 population, In the USA, this number rises to about 30 new cases per 100,000 population , while Australia and New Zealand report an even higher rate of about 60 new cases per 100,000 population (Long et al., 2023). Common risk factors for melanoma are ultraviolet (UV) radiation, number of abnormal nevi, and genetic background (Yang et al., 2018). Recently, immunotherapy has gained prominence as a treatment strategy for melanoma (Bagchi et al., 2021).

Bibliometric analysis refers to a quantitative approach that assesses significant research trends and focal points within the scientific community (Qi and Ren, 2023). Based on the assessment of database and literature characteristics, bibliometrics can uncover trends in academic publications and identify emerging frontiers of research. In addition, this method provides reliable data that can guide experimental methodologies and funding strategies (Liu et al., 2022). In the past few years, various bibliometric analyses connected to gut microbiota or melanoma have been published (Zhang et al., 2021; Deng et al., 2023). However, there has yet to be a comprehensive bibliometric analysis focused specifically on the role of gut microbiota in melanoma. Consequently, this study aims to conduct a thorough examination of research in this area to evaluate its current landscape and pinpoint key topics of interest.

## Methods

### Data sources and search strategies

In this study, the literature data source was compiled using Science Citation Indexing Expanded database from Web of Science, which encompasses over 10,000 influential journals and provides detailed citation data, widely utilized in academia (You et al., 2021). To minimize potential biases and account for the database's frequent updates, all articles were retrieved in 1 day (15 Feb 2025). The publication period in this study was set between 2014 and 2023. To determine the validity of the retrieval formula, we consulted the literature on gut microbiota (Chen Z. et al., 2023) as well as melanoma (Xu Y. et al., 2022). The search term was presented as follows: (((TS = (gut OR intestin^*^ OR gastrointestin^*^)) AND TS = (microbio^*^ OR microflora OR flora OR bacteri^*^ OR dysbiosis OR microecology OR 16Sr^*^ OR metagenome)) OR TS = (prebiotic^*^ OR probiotic^*^ OR synbiotic^*^)) AND (TS = (melanoma OR melanocarcinoma) OR TI = (melano^*^ OR melanoma OR melanocarcinoma) OR AB = (melano^*^ OR melanoma OR melanocarcinoma)). The operator AND can search for records containing all search terms separated by this operator, whereas the operator OR can search for records containing any search terms separated by this operator (Xu P. et al., 2022). And TS is means Topic, TI is means Title, AB is means Abstract. And our search method follows the prisma guide (Page et al., 2021).

### Inclusion and exclusion criteria

**Figure 1:**
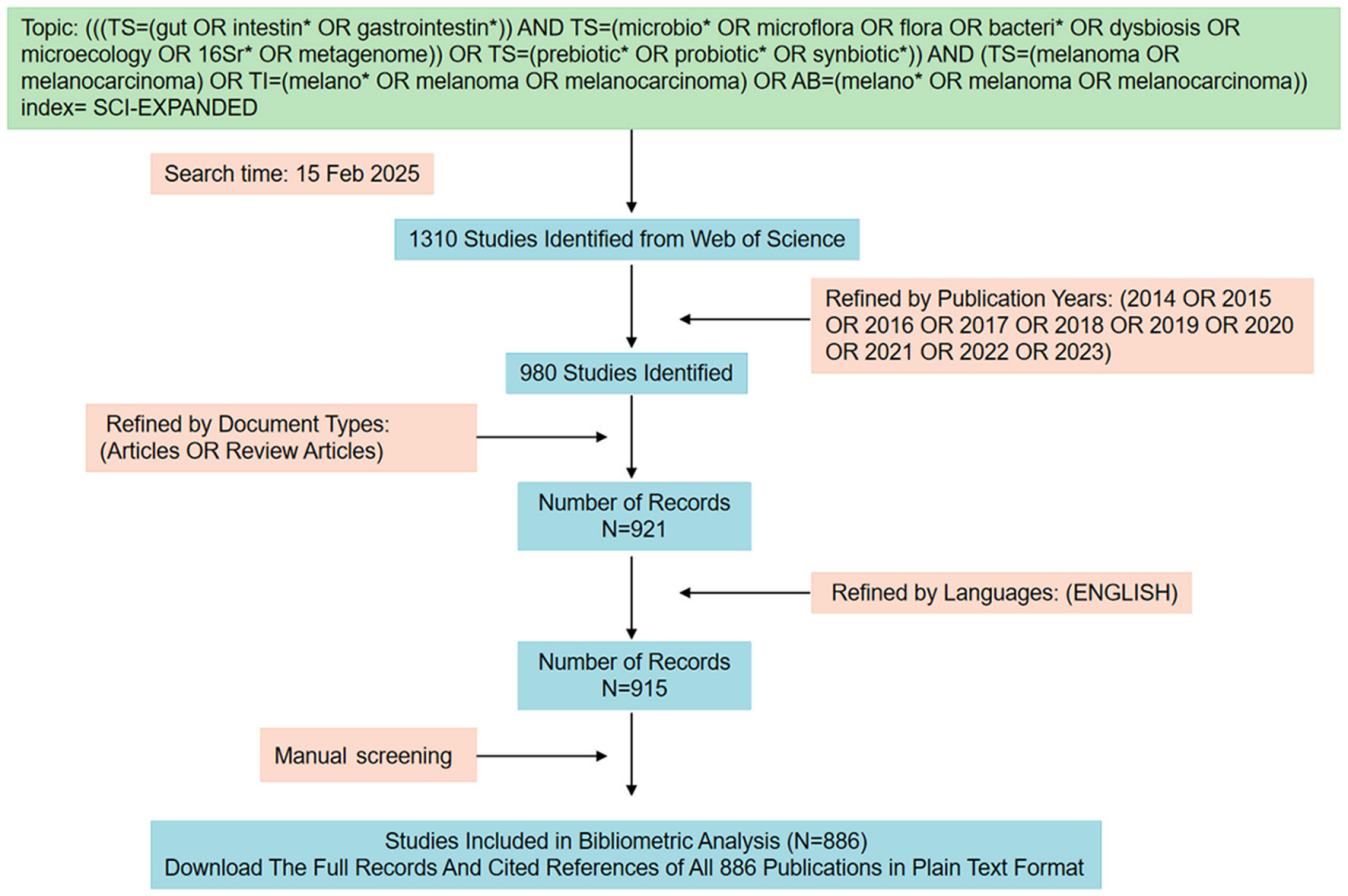
Flowchart of retrieval strategy.

Among the numerous publications, this study includes original articles and review papers written in English. Two authors (Sai Liu and Kewang Hu) independently screened the literature, and differences were resolved by consensus with the third author (Mingli Zhu). Articles that primarily examined or reported on gut microbiota and melanoma were considered. Papers unrelated to the subject of the study were routinely eliminated. The data were saved and stored in a download_txt format. Finally, a total of 886 articles, published between 2014 and 2023, were analyzed. The retrieval strategy is presented in a flowchart (Figure 1).

### Data extraction and cleaning

In all the collected articles, the data are sourced from the Science Citation Index Expanded (SCI-expanded) database. The search characteristics used for publications included authors, citations, publication year, affiliations, H-index, references, countries/regions, journals, and keywords. Bibliometric analysis (Ding et al., 2014) and network visualization were performed with an online program (http://www.bioinformatics.com.cn/), VOSviewer v1.6.10.0, and CiteSpace (version 6.3.1).

### Bibliometric analysis

The number of publications (Np) is typically used to measure a researcher's productivity, while the number of citations (Nc) can reflect their influence (Wei et al., 2022). The H-index is a metric used to assess a scholar's scientific impact and academic productivity, based on the number of papers published and the citations they have received. It is also employed to evaluate the scientific output of research institutions or teams (Hirsch, 2005). The Impact Factor (IF) is an indicator used to evaluate the influence of academic journals, primarily indicating how frequently articles published in a given journal are cited within the academic community. In essence, a higher IF signifies more frequent citations, reflecting greater influence in a specific academic field (Roldan-Valadez et al., 2019). Co-citation is a method used to analyze the relationship between academic papers by measuring how often two documents are cited together in the same article. Keyword co-occurrence analysis investigates the frequency of simultaneous appearances of multiple keywords in the literature, elucidating the interconnections among various research topics and highlighting key themes and emerging issues within a research field (Merigó et al., 2018). By utilizing VOSviewer (Eck and Waltman, 2014) and CiteSpace software, bibliometric maps were constructed to analyze co-occurrence and co-citation patterns in the literature, yielding more comprehensive results.

## Results

### Publication overview

**Figure 2:**
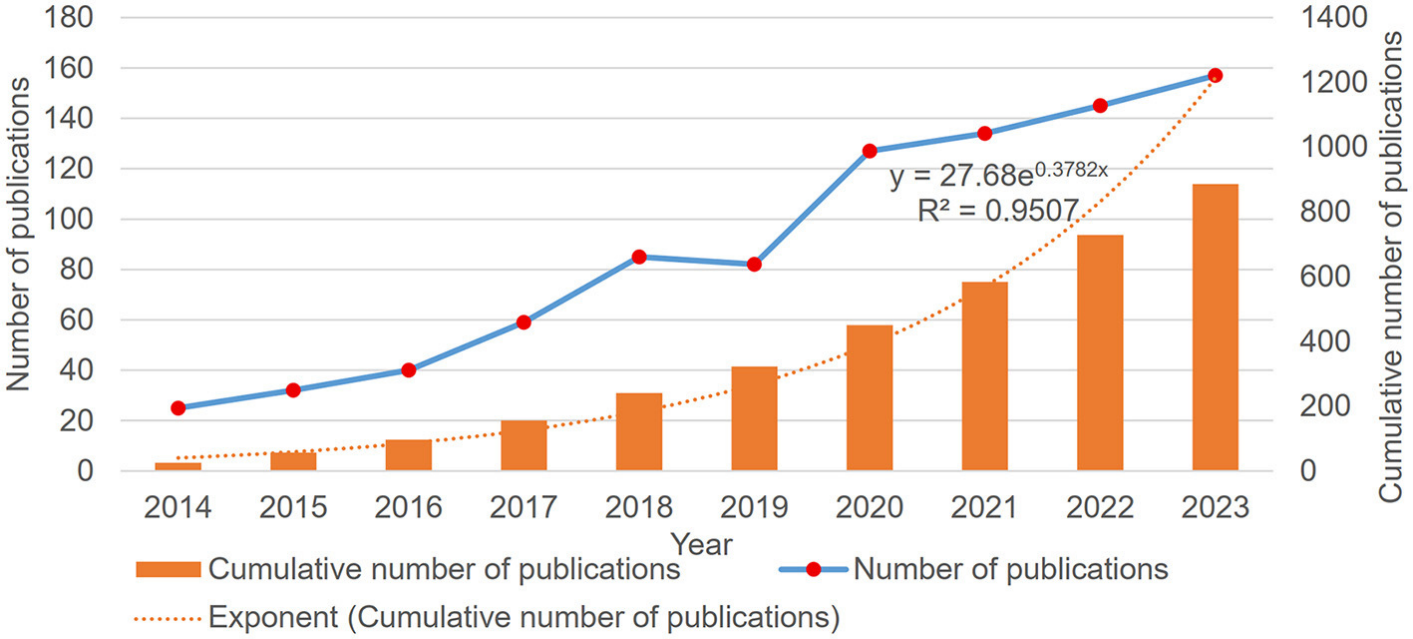
Number of publications per year over the past decade and exponential fit of the annual cumulative number of publications (*R*^2^ = 0.9103).

Based on our search strategy, 886 publications in the past 10 years were retrieved, including 668 original articles (75.40%) and 218 reviews (24.60%). Despite some fluctuations, the overall volume of literature on gut microbiota and melanoma showed a growth trend over the past decade (Figure 2). The exponential fit was used to represent the trend of the annual cumulative number of publications. The annual Np (the number of publications) trended upward (*R*^2^ = 0.9507). In summary, these findings indicate that research on the gut microbiome and melanoma has garnered significant attention and is developing rapidly.

### Analysis of countries/regions

**Figure 3:**
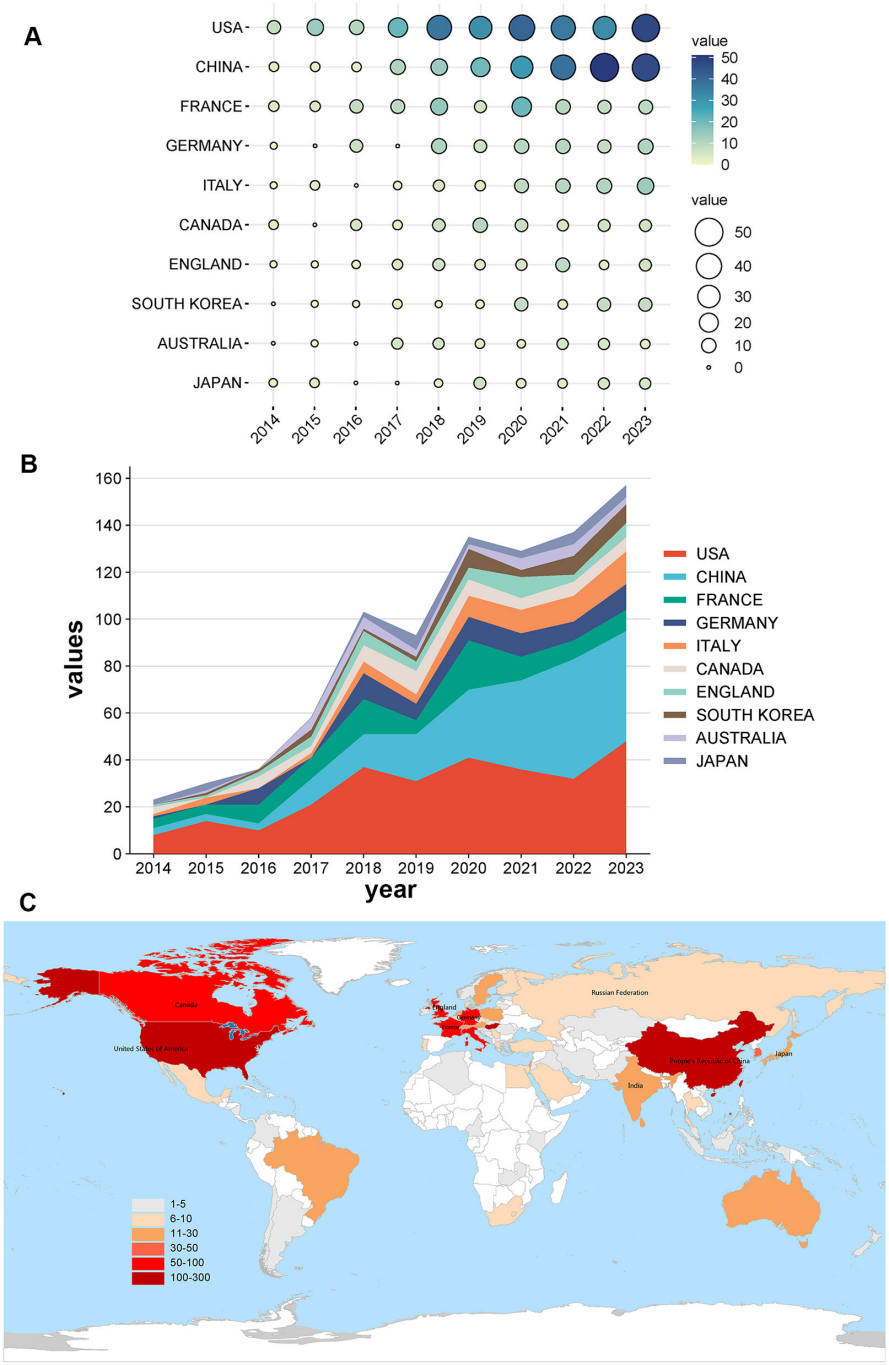
**(A-B)** Annual publication trends of the top 10 most productive countries (circle size and color representing the Np values of each country's output). **(C)** Publication counts distribution per country/region.

We ranked the top 10 countries/regions by Np based on the contributions of all authors (Table 1). Figures 3A, B illustrates the annual Np output of the most influential countries. Research on the role of gut microbiota in melanoma is at the forefront of global studies in the United States, while recent years have seen a surge in prolific research efforts in China. The participation of the countries/regions in the geographical distribution is shown in Figure 3C, with different colors represent different quantities of publications.

**Table 1:** The top ten countries/regions with the highest productivity.

**Rank**	**Country/Region**	**Np**	**Nc**	**H-index**
1	USA	278	33727	78
2	CHINA	219	8729	40
3	FRANCE	94	15312	33
4	GERMANY	65	3066	23
5	ITALY	56	4378	25
6	CANADA	52	5330	33
7	ENGLAND	41	3060	24
8	SOUTH KOREA	35	583	12
9	AUSTRALIA	29	1757	18
10	JAPAN	29	1786	13

Np, number of publications; Nc, number of citations.

**Figure 4:**
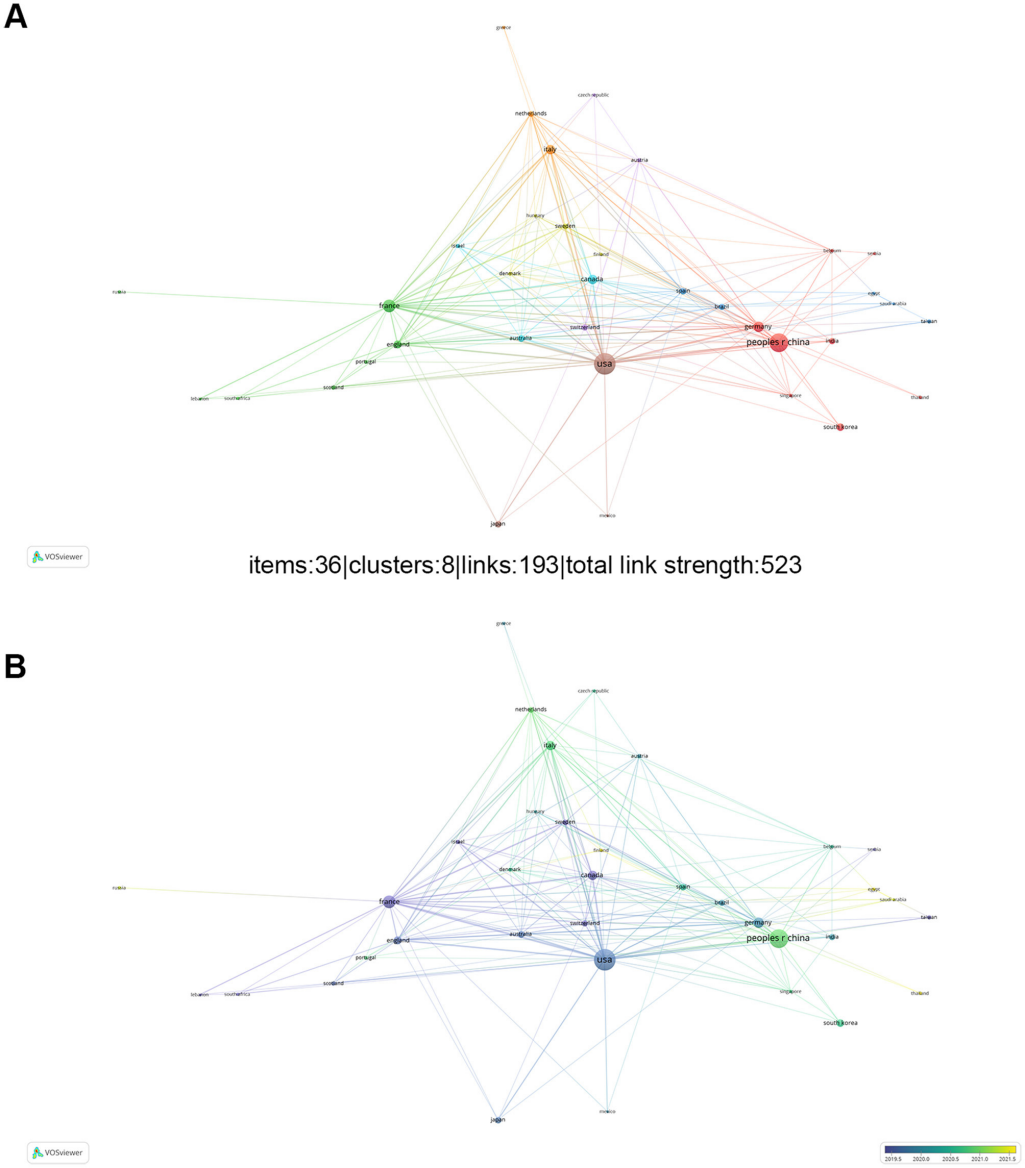
**(A)** The network of different countries visualization. Each node represents one country. circle size proportional to the number of publications. Connecting lines indicate collaboration between countries. Different colors represent different items. **(B)** Present the distribution of countries based on the average time of occurrence. Green and blue circles represent older publications, and yellow circles indicate more recent publications.

The publications were produced by 69 countries or regions, with 56 of them published more than two publications. In terms of Np, the United States ranked as the leading country (31.38%, 278/886), followed by China (23.81%, 211/886), and France (10.61%, 94/886). As for citations, American publications had been cited 33,727 times, representing 67.61% of total citations, followed by papers from France (15,312) and China (8,729). The United States (78), China (40), and France (33), Canada (33) ranked as the top four countries with the highest H-index, respectively. Furthermore, VOSviewer was used to analyze cooperation between countries or regions, and to generate visualization maps of international collaboration (Figures 4A,B). The USA, as the most prolific country, maintained the strongest international collaborations, followed by the China, France. While China's contributions have been particularly notable in recent years. In conclusion, with a notably greater number of papers and citations on the function of gut microbiota in melanoma than other nations, China, the US, and France are unquestionably at the forefront of this research. Additionally, they serve as key hubs for international collaboration.

### Analysis of affiliations

**Figure 5:**
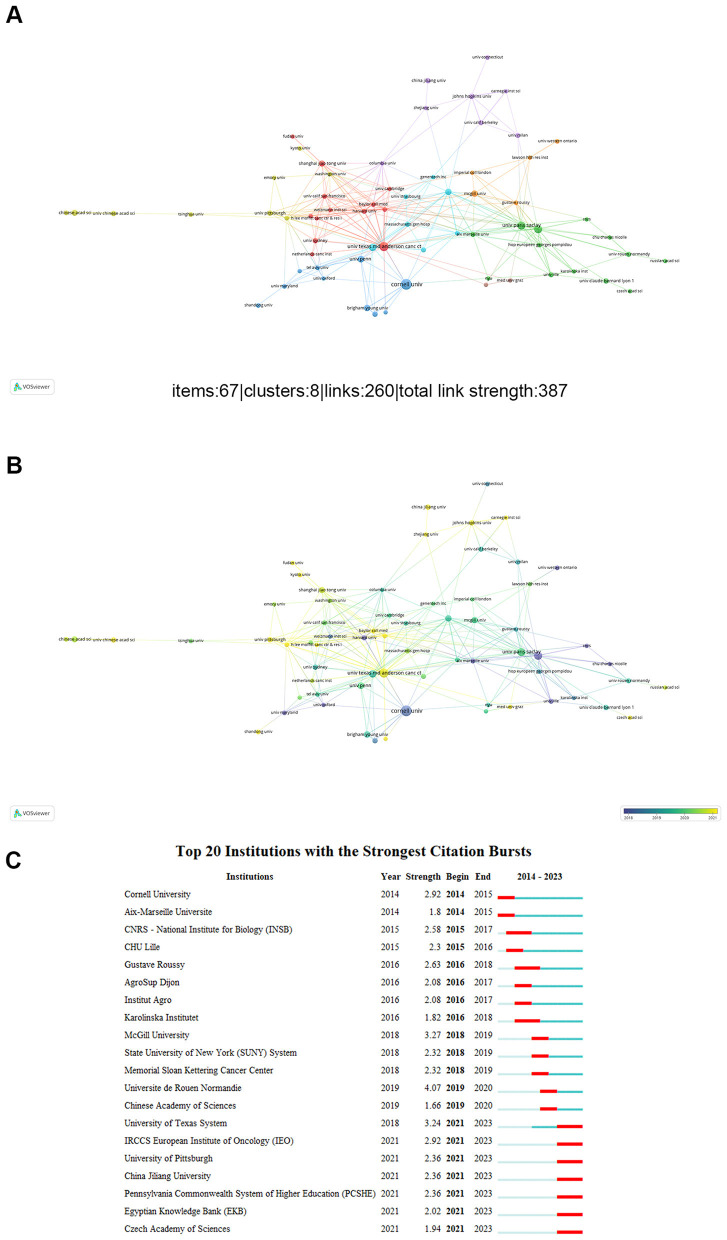
**(A-B)** Institutions' co-authorship network visualization map. **(C)** Top 20 institutions' co-authorship with strong occurrence bursts.

The ten most significant institutions, ranked by Np on the role of gut microbiota in melanoma, are presented in Table 2. The majority of these leading institutions are located in the United States and France. The greatest Np was achieved by INSERM (46), CNRS (44) is next, followed by the CORNELL UNIVERSITY (37). Meanwhile, INSERM achieved the highest Nc (13,821), while UNIVERSITY OF TEXAS SYSTEM had the greatest H-index (25). It suggests that these two countries have substantial influence in this research field. Figures 5A, B illustrates the institutional co-authorship network. The most collaborations are at the University of Texas MD Anderson Cancer Center, which is followed by Paris-Saclay University and INSERM. Figure 5C highlights the top 20 organizations with the most intense outburst. China Jiliang University, University of Pittsburgh, and IRCCS European Institute of Oncology (IEO) had the strongest burst values over the last 3 years. Overall, global research on the relationship between gut microbiota and melanoma is extensive and closely linked with numerous research centers.

**Table 2:** The top ten most productive affiliations.

**Rank**	**Affilistions**	**Np**	**Nc**	**H-index**	**Country**
1	INSTITUT NATIONAL DE LA SANTE ET DE LA RECHERCHE MEDICALE INSERM	46	13821	24	France
2	CENTER NATIONAL DE LA RECHERCHE SCIENTIFIQUE CNRS	44	9431	24	France
3	CORNELL UNIVERSITY	37	6522	24	USA
4	UNIVERSITY OF TEXAS SYSTEM	34	8681	25	USA
5	HARVARD UNIVERSITY	28	6398	23	USA
6	UNIVERSITY OF CALIFORNIA SYSTEM	26	2477	17	USA
7	INRAE	25	11713	16	France
8	UTMD ANDERSON CANCER CENTER	23	7757	18	USA
9	UNIVERSITE PARIS SACLAY	22	12886	15	France
10	CNRS NATIONAL INSTITUTE FOR BIOLOGY INSB	21	8362	15	France

H-index, H is for high citations.

### Analysis of authors

Based on the data retrieved, the ten most prolific and active authors are listed in Table 3. Douglas, Angela E (Cornell University) was the largest output writer, (Np = 19) with the greatest H-index (15). Followed by Wargo, Jennifer A (University of Texas System), and Chaston, J. M (Brigham Young University). In addition, Zitvogel, Laurence (Universite Paris Saclay) had the highest Nc (11,501). The research conducted by Zitvogel, Laurence has garnered more interest from academics.

**Table 3:** The top ten authors with the most publications.

**Rank**	**Author**	**Affiliations**	**Country**	**Np**	**Nc**	**H-index**
1	Douglas, Angela E.	Cornell University	USA	19	1155	15
2	Wargo, Jennifer A.	University of Texas System	USA	14	7499	12
3	Chaston, J. M.	Brigham Young University	USA	14	547	10
4	Routy, Bertrand	Universite de Montreal	Canada	13	8605	13
5	Leulier, Francois	Center National de la Recherche Scientifique	France	12	347	9
6	Newell, P. D.	Brigham Young University	USA	12	693	11
7	Zitvogel, Laurence	Universite Paris Saclay	France	11	11481	10
8	Fetissov, Sergueï O	Universite de Rouen Normandie	France	10	384	9
9	N. Buchon	University of Florida	USA	8	543	6
10	Wong, Adam Chun-Nin	Cornell University	USA	8	567	8

### Analysis of journals

The most studies were published by Frontiers in Immunology (28 publications, IF: 7.3), as indicated in Table 4, followed by Scientific Reports (22 publications, IF: 4.6) and Cancers (22 publications, IF: 5.2). In addition, the journal with the biggest influence was Science (Nc = 15,563, H-index = 10, IF: 56.9), it determines that this field is well worth studying.

**Table 4:** The top twelve most-published journals.

**Rank**	**Journal**	**Np**	**Nc**	**H-index**	**IF (2022)**
1	FRONTIERS IN IMMUNOLOGY	28	1101	15	7.3
2	SCIENTIFIC REPORTS	22	558	13	4.6
3	CANCERS	22	427	11	5.2
4	INTERNATIONAL JOURNAL OF MOLECULAR SCIENCES	18	284	8	5.6
5	PLOS ONE	16	432	11	3.7
6	APPLIED AND ENVIRONMENTAL MICROBIOLOGY	16	716	12	4.4
7	MICROORGANISMS	13	165	8	4.5
8	NUTRIENTS	12	444	10	5.9
9	ISCIENCE	11	110	6	5.8
10	MBIO	11	785	10	6.4
11	SCIENCE	10	15545	10	56.9

IF, impact factor.

### Analysis of article global citations

**Figure 6:**
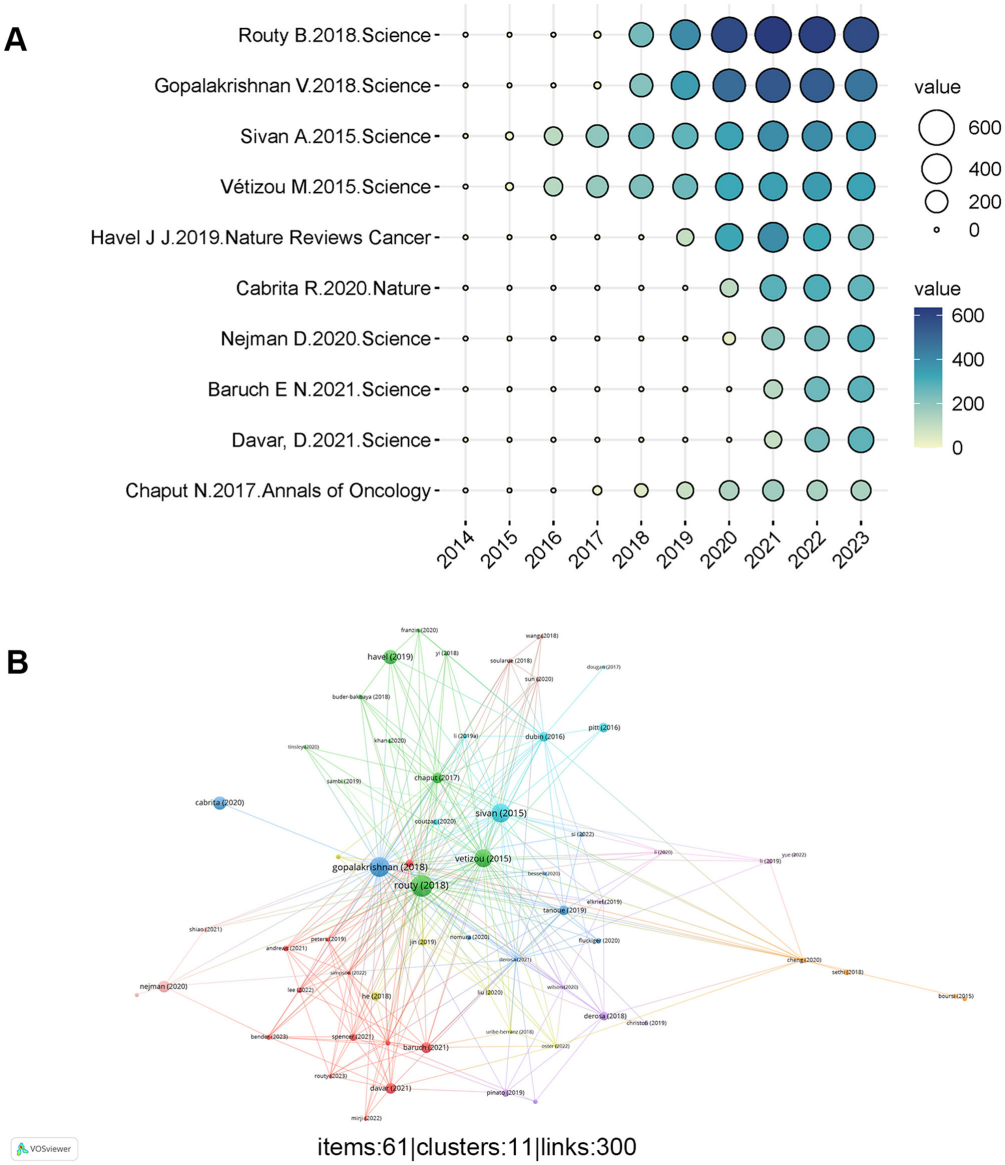
**(A)** The annual quantity of global citations for papers having a high global citation score (the circle's size and color indicate the global citation score) **(B)** Network of document citation. Given a large number of references available, the minimum number of citations for a reference was placed at 100. Out of the 914 publications, 74 were selected for citation analysis. The different colors of the nodes represent different documents, with larger nodes meaning more frequently cited articles.

The papers in Table 5 are listed in descending order based on the total number of citations. Most of the top 10 cited papers were published in prestigious journals such as Nature and Science. Figure 6A illustrates the annual number of global citations for articles with a high Global Citation Score (GCS). And most of the referenced articles emphasize the critical influence of human gut commensal microbes on melanoma immunotherapy. There are complex interactions between melanoma patients' response to cancer immunotherapy and the intestinal flora which may help shape improved immunotherapy strategies in the future. Figure 6B displays 92 highly referenced articles that received more than 100 citations from other publications, according to Figure 6A, where the center of the mesh is made up of relatively large nodes representing articles with high GCS.

**Table 5:** The top ten highest cited articles.

**Rank**	**Year**	**Artical**	**IF (2022)**	**Total citation**	**Type of study**
1	2018	Routy B, Le Chatelier E, Derosa L, et al. Gut microbiome influences efficacy of PD-1-based immunotherapy against epithelial tumors[J]. Science (New York, NY), 2018, 359(6371): 91-97.	56.9	3659	Article
2	2018	Gopalakrishnan V, Spencer C N, Nezi L, et al. Gut microbiome modulates response to anti-PD-1 immunotherapy in melanoma patients[J]. Science (New York, NY), 2018, 359(6371).	56.9	3,102	Article
3	2015	Sivan A, Corrales L, Hubert N, et al. Commensal Bifidobacterium promotes antitumor immunity and facilitates anti-PD-L1 efficacy[J]. Science (New York, NY), 2015, 350(6264): 1084-1089.	56.9	2,717	Article
4	2015	Vétizou M, Pitt J M, Daillère R, et al. Anticancer immunotherapy by CTLA-4 blockade relies on the gut microbiota[J]. Science (New York, NY), 2015, 350(6264): 1079-1084.	56.9	2,448	Article
5	2019	Havel J J, Chowell D, Chan T A. The evolving landscape of biomarkers for checkpoint inhibitor immunotherapy [J]. Nature Reviews Cancer, 2019, 19(3): 133-150.	78.5	1,625	Review
6	2020	Cabrita R, Lauss M, Sanna A, et al. Tertiary lymphoid structures improve immunotherapy and survival in melanoma [J]. Nature, 2020, 577(7791): 561-565.	64.8	1,334	Article
7	2020	Nejman D, Livyatan I, Fuks G, et al. The human tumor microbiome is composed of tumor type-specific intracellular bacteria[J]. Science (New York, NY), 2020, 368(6494): 973-980.	56.9	1,130	Article
8	2021	Baruch E N, Youngster I, Ben-Betzalel G, et al. Fecal microbiota transplant promotes response in immunotherapy-refractory melanoma patients[J]. Science (New York, NY), 2021, 371(6529): 602-609.	56.9	908	Article
9	2021	Davar D, Dzutsev A K, Mcculloch J A, et al. Fecal microbiota transplant overcomes resistance to anti-PD-1 therapy in melanoma patients[J]. Science (New York, NY), 2021, 371(6529): 595-602.	56.9	895	Article
10	2017	Chaput N, Lepage P, Coutzac C, et al. Baseline gut microbiota predicts clinical response and colitis in metastatic melanoma patients treated with ipilimumab[J]. Annals of Oncology : Official Journal of the European Society For Medical Oncology, 2017, 28(6): 1368-1379.	50.5	885	Article

**Figure 7:**
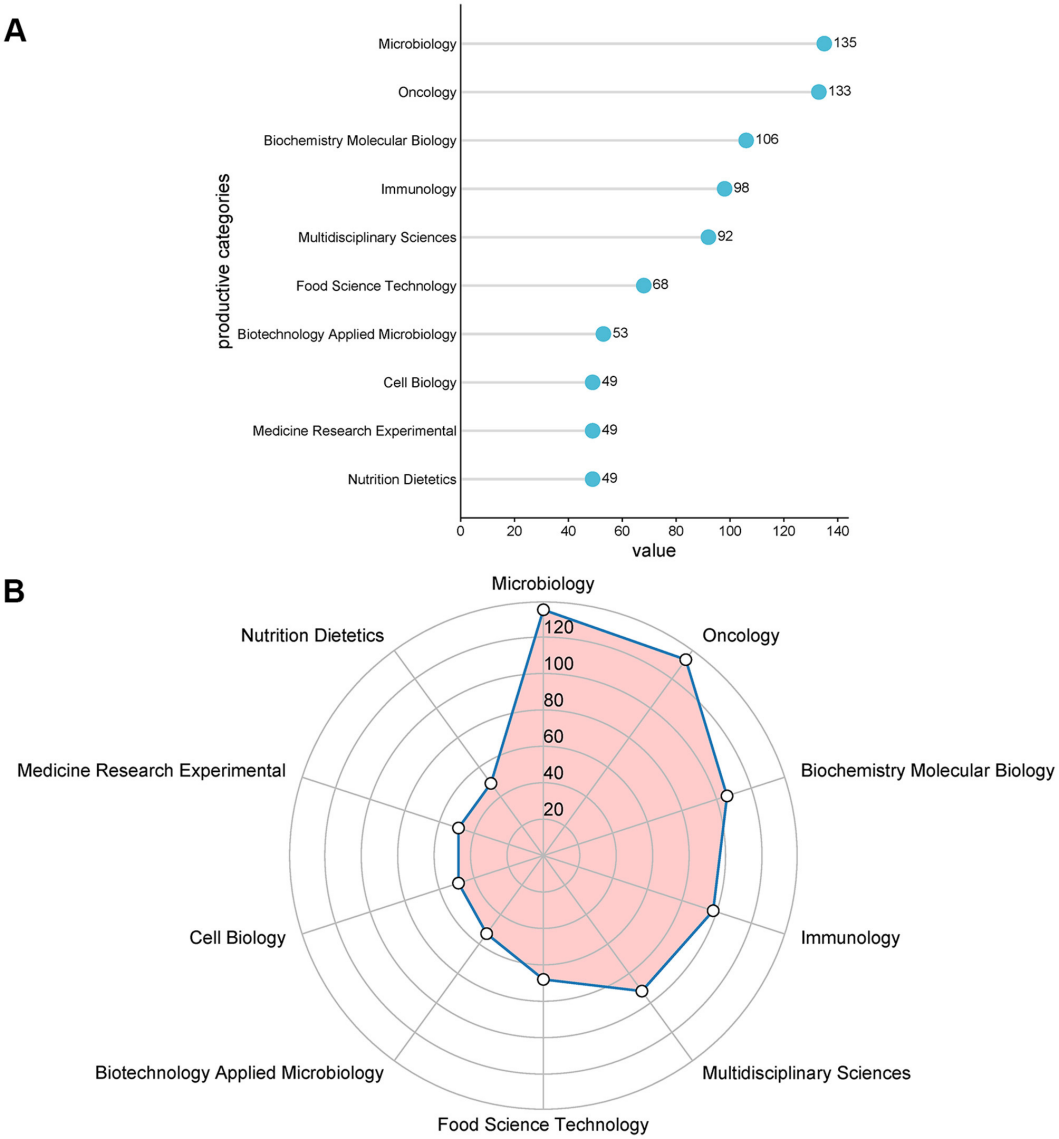
**(A-B)** The top ten research productive categories.

The top 10 productive categories linked to the role of gut microbiota in melanoma are shown in Figures 7A,B. Microbiology (135 papers), Oncology (133 papers), and Biochemistry Molecular Biology (106 papers) were the most common study categories.

### Analysis of co-cited references

**Figure 8:**
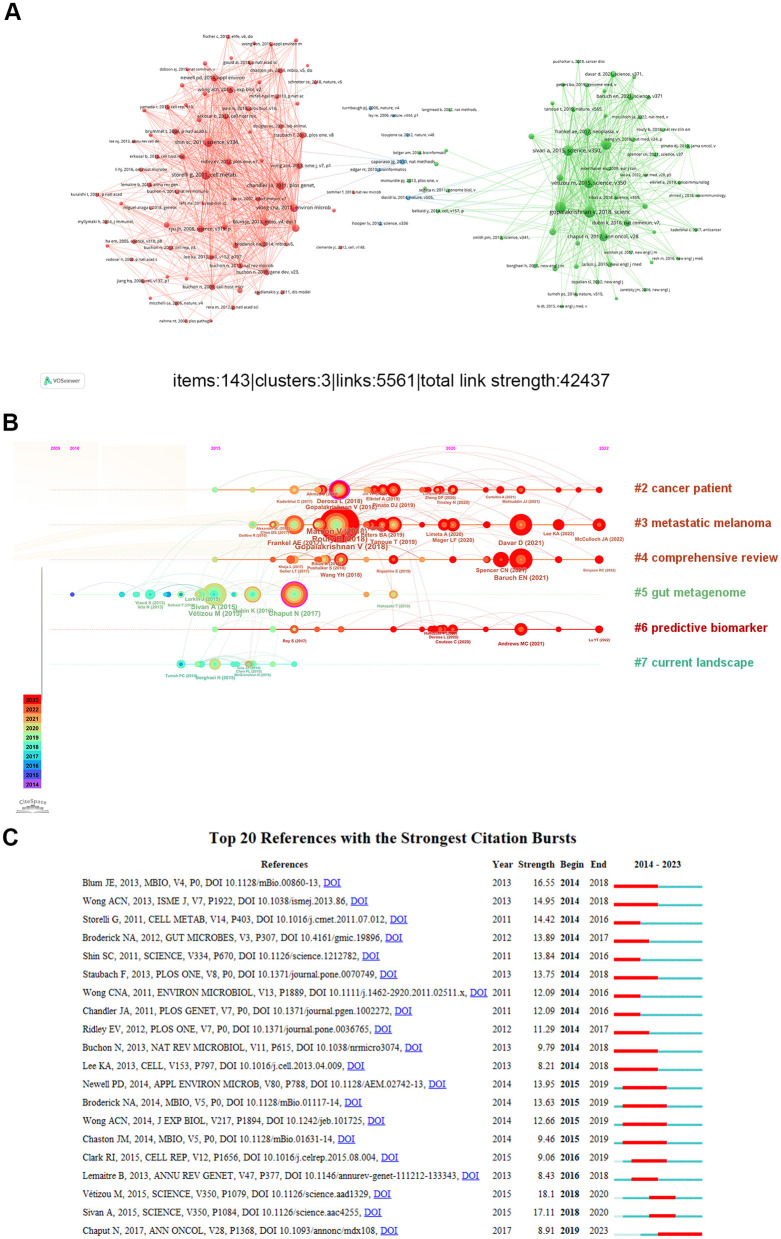
**(A)** A network diagram of co-cited references. **(B)** The top eight clusters' timeline distribution. **(C)** The top 20 co-cited references with the most citation burstiness.

In contrast to global citation analysis, co-citation networks emphasize research topics that are closely associated with specific fields. Due to the large volume of references, a minimum of 20 citations was required for a document to be included. Out of the 43,957 references cited by the articles that were retrieved, 143 were selected for co-citation analysis. Two nodes that are connected by a line show that they were mentioned in the same publication, a shorter line suggests a closer relationship between two publications. Additionally, the papers were divided into clusters using various node colors (Figure 8A). Cluster 1 (red), which includes 79 references, is centered on the function of drosophila gut microorganisms, cluster 2 (green) included 57 references focused primarily on the function of gut microbiota in melanoma immunotherapy, Cluster 3 (blue) contained 7 references mainly focused on how gut flora and obesity are related. Figure 8B shows the most common references for burst time, burst strength, and burst duration. We found these clustering tags are not continuous, show that the co-occurrence network did not form a large connected network, but some small unconnected networks, and cite space only shows the largest network. This also indicates that there are few studies in this field and it is a topic direction worthy of further mining subject direction. The top 20 co-cited references with a high incidence burst in the research on the intestinal microbiota in melanoma are displayed in Figure 8C. Vétizou M's article from 2015 about the CTLA-4 blocking as a cancer immunotherapy relies on the gut microbiota that had the strongest occurrence burst.

### Analysis of hotspots in research

**Figure 9:**
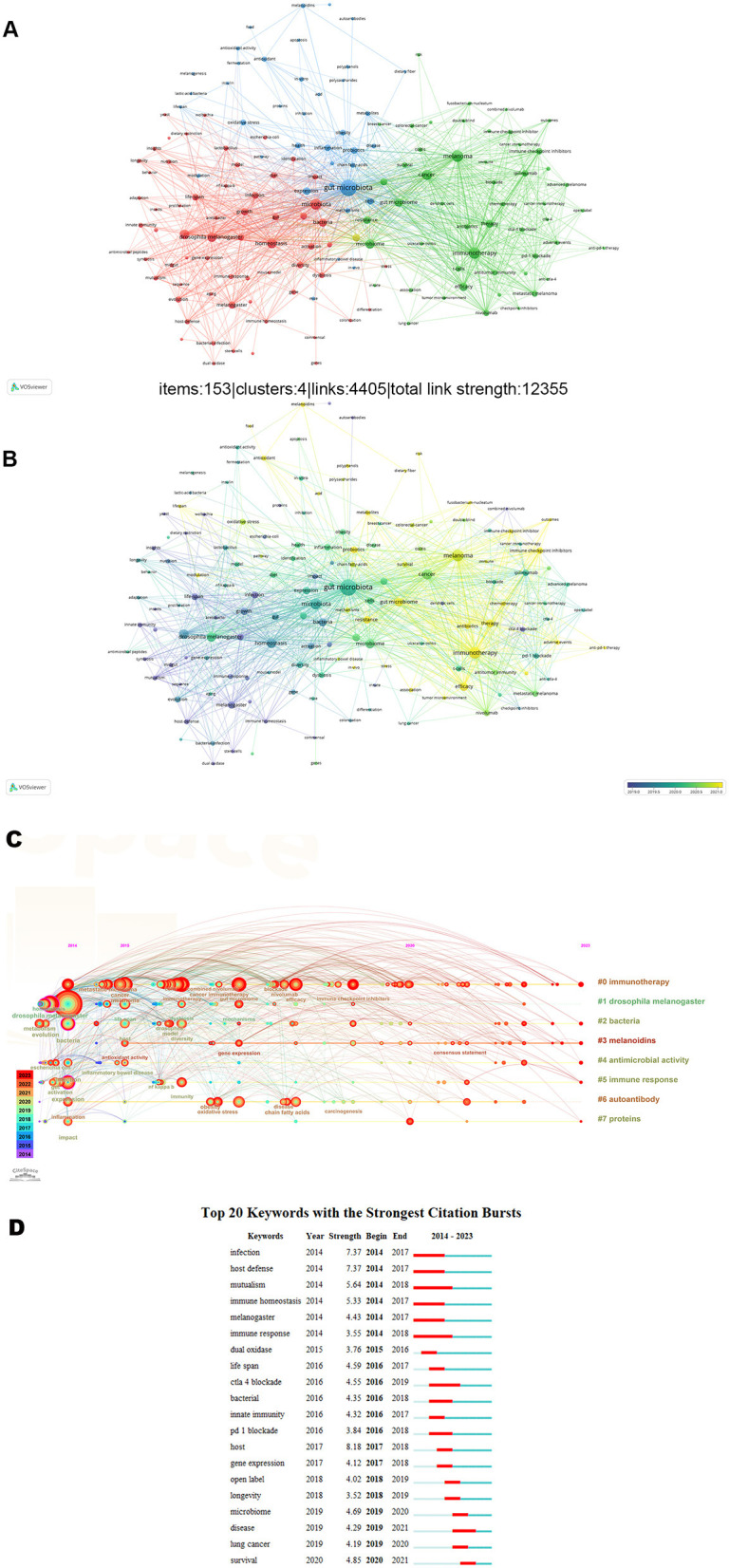
**(A-B)** A network diagram of keywords. **(C)** Timeline distribution of keyword cluster analysis. **(D)** The top twenty keywords with the most bursts.

The purpose of keyword co-occurrence analysis is to examine the link between keywords that appear together in a collection of publications that may represent popular subjects. A total of 4,281 keywords appeared in 886 publications, and 153 of them appeared more than 10 times. We divided these keywords into four clusters and marked them with different colors. The node's size indicates how frequently an event occurs. As seen in Figure 9A, cluster 1 (red) is mostly concerned with the role of gut microbes in drosophila melanogaster, cluster 2 (green) reflected the basic characteristics of gut microbes, cluster 3 (blue) primarily concerned with the function of gut flora in immunotherapy for melanoma and cluster 4 (yellow) concentrated on melanoma and gut microbial immunity. Figure 9B shows a visualization of keywords based on the typical publishing year. The various hues denote the pertinent year of release. and keywords in yellow are more recent than those in purple. This result indicated that hotspots for research are currently centered on how the gut microbiota affects immunotherapy for melanoma. The timeline image can display the duration and temporal trend of various research keywords. As seen in Figure 9C, immunotherapy, drosophila melanogaster, bacteria, melanoidins, antimicrobial, immune response, autoantibody and proteins, are the longest-lasting hotspots. Keyword occurrence analysis can find the keywords with higher frequency change rate in the corresponding time node, and judge the frontier of research focus. We show the top 20 keywords with the most powerful bursts of citations in Figure 9D.

## Discussion

The relationship between gut microbiota and tumors represents a significant area of investigation in contemporary cancer research (Park et al., 2022). Gut microbiota not only play a crucial role in sustaining the host's metabolic and immune balance, but also may play a major part in the occurrence, progression and treatment of tumors (Li et al., 2019). Melanoma, a highly aggressive form of skin cancer (Schadendorf et al., 2018), has attracted increasing attention in recent years for its potential connection with intestinal bacteria in both its development and treatment (Gopalakrishnan et al., 2018). Nowadays, more researchers have employed bibliometric analysis to examine the status and trends within specific scientific fields (Kokol et al., 2021). Whereas, no bibliometric study has yet investigated the impact of gut microbiota on melanoma. Therefore, this will be the first study to explore the relationship between gut flora and melanoma predisposition, potentially offering valuable insights for future research.

A total of 886 articles and reviews published between 2014 and 2023 were analyzed. Although the number of publications fluctuated slightly over the past decade, there was a steady increase in research on melanoma and the gut microbiota (Figures 2A,B). Over the past decade, the United States was the most productive nation in this field, followed by China, which, despite starting later, has rapidly advanced (Figures 3A,B). Significantly outperforming other countries, China and the USA both showed a steadily rising trend in annual publications. Although France produces fewer articles than China, its research has a broader influence (Table 1). In light of this, Chinese scholars and institutions should prioritize enhancing the quality of their research to improve its global impact. Throughout the period from 2014 to 2023, publications are distributed globally, although research productivity remains modest in many regions (Figure 3C). Furthermore, effective international cooperation promotes academic exchanges and cooperation. The network map emphasizes the substantial collaboration and central positioning of China and the United States in the multi-country collaboration network (Figure 4A). China's cooperation has been particularly significant in recent years (Figure 4B).

In terms of institutional affiliations (Table 2), nearly all of the top ten organizations in this field were based in the United States or France. The top 10 affiliations and authors included five US institutions and six US researchers (Table 3), demonstrating the country's superior academic institutions and specialized knowledge, which has contributed to its dominance in this field over the previous 10 years. Additionally, French institutions and scholars exert substantial influence. Therefore, to remain informed about the latest advancements in this field, it is essential to prioritize and pay closer attention to their contributions. According to the network of connections between institutions, the UTMD Anderson Cancer Center was situated in the hub of these collaborations (Figures 5A,B). Douglas, Angela E. from Cornell University generated the largest volume of publications (Np = 19), with his team maintaining a long-term research focus on the functions and mechanisms of gut microbes in drosophila. Followed by Wargo, Jennifer A (Np = 14) from University of Texas System, Wargo's team elucidated the role of microbes in cancer and the mechanisms underlying their therapeutic effects. Additionally, they have also undertaken extensive investigations into the functions and mechanisms of gut microbiota in the context of melanoma. For instance, in several highly cited articles, the gut microbiome influences how melanoma patients respond to anti-PD-1 immunotherapy (Gopalakrishnan et al., 2018). Tertiary lymphoid structures are crucial within the immune microenvironment of melanoma, suggesting that exploring therapeutic approaches to promote their formation could enhance the efficacy of cancer immunotherapy (Cabrita et al., 2020). Wargo's latest study investigated that antibiotic conditioning alters the gut microbiota and immune function in patients with metastatic melanoma who are not undergoing immune checkpoint inhibition (Glitza et al., 2024). This unique, randomized, placebo-controlled, biomarker-driven microbiome modification experiment showed that the combination of vancomycin, SER-401, and anti-PD-1 therapy is safe for melanoma patients. Additionally, it suggests that while planning such studies, antibiotic preconditioning and interventional medication dosing regimens should be carefully taken into account.

Notably, A high IF (IF >5) was found in seven of the top eleven most prolific journals (Table 4). In particular, ten articles were published in science. It seems to imply that publishing research in this field within prestigious journals does not pose significant difficulty. Meanwhile, this finding suggested that Science had released some potentially ground-breaking findings about the role of gut microbiota in melanoma. It also serves as a reminder to scholars who are interested in this area to think about submitting their work to these journals.

Of the ten most cited articles (Table 5), 90% (9/10) focused on the impact of gut microbiota on melanoma immunotherapy, indicating that this research direction is a highly active area of interest among scholars. Only Deborah's research (Nejman et al., 2020) was not focused on the immunotherapy side, they performed an extensive investigation into the tumor microbiome, identifying unique microbial compositions associated with each specific type of tumor. Additionally, researchers found associations between intratumor microorganisms or their expected roles and the different types and subtypes of tumors, the smoking status of the patients, and the patients' reaction to immunotherapy. Routy B, Gopalakrishnan V, Sivan A, and Vétizou M are the four researchers whose works have received more than 2,000 citations. Routy B's 2018 article was the most cited, with 3659 citations, securing the top position (Figures 6A,B). His studies revealed that initial resistance to ICIs is influenced by the gut microbiome's abnormal composition. In individuals with advanced cancer, antibiotic treatment may reduce the therapeutic benefit of immune checkpoint inhibitors. Burst detection results showed that 2018 was a breakout point, with a large number of high-quality articles published. Research has revealed that modulation of the gut flora may help improve the response of cancer patients to PD-1 blockade, especially after the administration of antibiotics (Routy et al., 2018). Consequently, this work received the highest number of citations, reflecting its significant recognition by other scholars. What's more, Gopalakrishnan V discovered that melanoma patients' reaction to anti-PD-1 therapy is correlated with the composition of their gut flora, with those possessing a richer and more diverse microbiome demonstrating a better response to immunotherapy (Gopalakrishnan et al., 2018). Matson analyzed the microbiomes of baseline fecal samples from patients with metastatic melanoma and found a significant correlation between the abundance of specific bacterial species and the patients' responses to anti-PD-1 immunotherapy. These findings suggest that the symbiotic microbiome could serve as a biomarker for predicting immunotherapy responses and influence anti-tumor immune responses (Matson et al., 2018). The findings open the possibility of developing new therapeutic strategies, such as microbial transplantation or probiotics to improve melanoma patients' response to immunotherapy. In a similar vein, the studies of Sivan A revealed that the Intestinal commensal bacterium bifidobacterium enhances dendritic cell function by promoting the development and accumulation of CD8+ T lymphocytes in the tumor microenvironment, promoting anti-tumor immunity and enhancing anti-PD-L1 treatment's effectiveness (Sivan et al., 2015). Additionally, Vétizou M found that in some patients, altering the makeup of the gut flora may improve how well CTLA-4 blocking works, suggesting potential avenues for developing novel therapeutic strategies (Vétizou et al., 2015).

Research frontiers and trends can be explored with the aid of hot spot analysis. The “immune response” and “PD-1” hotspots of constant concern were shown by co-cited references and keywords displayed chronologically. We found from the clustering that most studies concentrated on the basic therapeutic and diagnosis studies. Co-occurrence is a tool used to assess the relationship between recorded items. From the analysis of the keyword map, “gut microbiota”, “immunotherapy” and “melanoma” are positioned centrally. Based on this, the trendy terms that have emerged within the previous 4 years are “genes”, “survival”, “lung cancer”, “microbiome”, “longevity”. It can be seen from this that in the previous 4 years, research on the function of intestinal flora in melanoma spans from immune mechanisms to extending survival in clinical immunotherapy, and has expanded from melanoma to other types of cancer.

Through this study, we found that, over time, the major players in the research field gradually expanded from traditional western developed countries, such as the United States and France, to emerging developing countries such as China. This change not only reflects the trend of Sino-Western cooperation to promote research progress, but also reflects the rapid catch-up and contribution of researchers from emerging countries in this field. In particular, there has been remarkable progress in modulating the efficacy of immune checkpoint inhibitors, such as anti-PD-1/PD-L1 therapies. The latest research also proposed an innovative gut chip model, which can integrate stool samples of patients and predict the effect of immunotherapy, thus providing a new tool for individualized treatment (Ballerini et al., 2025). Our study shows that the gut microbiota, as an adjustable immunomodulatory factor, has opened up new avenues for precision treatment of melanoma. In the future, the combination of multi-omics technologies (such as metagenomics and metabolomics) and organoid models will help to better understand the microbiota-host interaction network and promote the clinical application of microbial-targeted therapies.

### Moving the filed forward

Over the past decade, immune checkpoint inhibitors (ICIs) have significantly improved the survival rate of melanoma patients, but their efficacy is regulated by intestinal microorganisms. Future research needs to deeply analyze the mechanism of intestinal microbial-immune interaction and further clarify how intestinal microorganisms regulate T cell activation and dendritic cell (DC) maturation through metabolites (such as short-chain fatty acids, tryptophan derivatives) or immune signaling molecules (such as IL-12, IFN-γ) (Routy et al., 2018). At the same time, bacterial transplantation technology should be optimized, long-term cohort research should be carried out, the association between dynamic changes in the microbiome and long-term treatment prognosis should be tracked, and a prediction model based on the microbiome should be established (Chaput et al., 2017). In terms of optimized treatment, microbial functional gene expression can be comprehensively evaluated through metagenomic sequencing and metabolomics, identify key bacterial species related to anti-tumor immunity, and detect the impact of microbial-derived metabolites on immune cell function. In addition, randomized controlled trials (RCTs) were conducted to further verify the safety and effectiveness of fecal bacteria transplantation (FMT) or probiotics combined with ICIs (Baruch et al., 2021). In terms of treatment strategy selection, individualized treatment plans can be formulated based on the patient's baseline microbiome characteristics (such as alpha diversity, key bacterial species abundance), FMT transplant responder microbiota for patients with ICIs resistant to restore anti-tumor immunity, or enhance intestinal barrier function and regulate immune responses by supplementing specific strains (Shao et al., 2017) or prebiotics (Bibbò et al., 2022). In terms of specific methods of manipulating the microbiota, FMT efficacy can be improved through strict screening of donors and optimized transplantation programs, avoiding the use of broad-spectrum antibiotics to protect beneficial bacteria, and recommending dietary fiber-rich diets to promote the proliferation of butyrate-producing bacteria (Gopalakrishnan et al., 2018). For the recommendation of pharmacological regulation of microbiota, patients with ICIs-refractory melanoma can try FMT as a combination treatment method (Davar et al., 2021), but closely monitor the risk of infection and the occurrence of enteritis. At the same time, microbiome detection is included in the melanoma treatment decision-making process, and precise treatment is achieved by combining genome, transcriptome and metabolomic data.

### Strengths and limitations

This study makes use of bibliometric and bibliographic visualization studies of the literature, which could aid scholars in comprehending the field's advancement tendencies and scholarly boundaries. Previous studies have employed bibliometric analysis to investigate the relationship between the gut microbiome and various cancers, including colorectal (Wu W. et al., 2023), lung (Chen H. et al., 2023), liver (Chen Z. et al., 2023), gastric (Zhang et al., 2020), and pancreatic cancers (Wu S. et al., 2023). To our knowledge, this is the first bibliometric analysis of the role of gut microbiota in melanoma. Additionally, we used the WoSCC database to identify relevant literature on the function of intestinal flora in melanoma over the past decade, which may assist scholars in identifying key trends in this sector. WoSCC is widely recognized as a representative source of high-quality scientific literature due to its rigorous journal selection criteria (based on impact, quality of peer review, etc.) (Garfield, 2006). This study focused on the process of mainstream academic consensus formation, so this database was preferentially selected. WoSCC provides standardized metadata and citation network tracking tools (e.g., citation reports, subject classification codes) for interdisciplinary trend analysis and knowledge mapping construction (Mongeon and Paul-Hus, 2016). And structured data provided by WoSCC supported our analysis. However, this research has certain restrictions. First, included were only reviews and articles written in English from SCI Expanded-indexed publications. WoSCC is mainly published in English, it may miss regionally important research results (such as some non-English journals) or content from emerging open access platforms. In addition, there is usually a delay of several months to a year between publication and inclusion in WoSCC. Therefore, the results of this study reflect more mid—and long-term research hotspots rather than real-time trends. To reduce the impact of bias, we manually searched key authors and institutional outputs to verify the robustness of the core findings. Future studies can integrate multi-source databases such as Scopus and Pubmed to enhance coverage. Second, the incapacity of Citespace and VOSviewer to evaluate published texts in their entirety may result in the omission of some details.

## Conclusion

This research used bibliometric analysis to perform a thorough quantitative and qualitative evaluation of the literature concerning the role of gut microbiota in melanoma, spanning the period from 2014 to 2023. It highlights the progression of publications and citation patterns in this field over the last ten years. The involvement of intestinal microbiota in melanoma initiation, progression, and treatment is gradually being revealed, particularly with promising applications in enhancing the efficacy of immunotherapy. The continued progress of research holds promise for improving melanoma treatment via controlling the intestinal microbiome, potentially leading to better patient outcomes in the future.

## Data Availability

The original contributions presented in the study are included in the article/supplementary material, further inquiries can be directed to the corresponding author.
