# Supplementary material for: Global research trends on the relationship between gut microbiota and melanoma from 2014 to 2023: a bibliometric and visualization analysis
Source: Front Microbiol. 2025 Aug 13;16:1524462. doi: 10.3389/fmicb.2025.1524462 (PMC12380698; doi:10.3389/fmicb.2025.1524462)
Supplement: Supplementary file 3 [file Supplementary_file_1.docx]

**Identification of studies via databases and registers**

Topic: (((TS=(gut OR intestin* OR gastrointestin*)) AND TS=(microbio* OR microflora OR flora OR bacteri* OR dysbiosis OR microecology OR 16Sr* OR metagenome)) OR TS=(prebiotic* OR probiotic* OR synbiotic*)) AND (TS=(melanoma OR melanocarcinoma) OR TI=(melano* OR melanoma OR melanocarcinoma) OR AB=(melano* OR melanoma OR melanocarcinoma)) index= SCI-EXPANDED

1310 Studies Identified from Web of Science

**Identification**

980 Studies Identified

Refined by Publication Years: (2014 OR 2015 OR 2016 OR 2017 OR 2018 OR 2019 OR 2020 OR 2021 OR 2022 OR 2023)

Number of Records

N=921

Refined by Document Types: (Articles OR Review Articles)

**Screening**

Refined by Languages: (ENGLISH)

Number of Records

N=915

Refined by manual screening

Number of Records

N=886

Studies included in review

(n = 218)

Reports of included studies

(n = 668)

**Included**

Source: Page MJ, et al. BMJ 2021;372:n71. doi: 10.1136/bmj.n71.

This work is licensed under CC BY 4.0. To view a copy of this license, visit <https://creativecommons.org/licenses/by/4.0/>
